# Body Roundness Index and All-Cause Mortality Among US Adults

**DOI:** 10.1001/jamanetworkopen.2024.15051

**Published:** 2024-06-05

**Authors:** Xiaoqian Zhang, Ning Ma, Qiushi Lin, Kening Chen, Fangjieyi Zheng, Jing Wu, Xiaoqun Dong, Wenquan Niu

**Affiliations:** 1 Graduate School, Beijing University of Chinese Medicine, Beijing, China; 2 Department of Pediatrics, China-Japan Friendship Hospital, Beijing, China; 3 Department of Pediatrics, Beijing University of Chinese Medicine Third Affiliated Hospital, Beijing, China; 4 Sanofi Aventis, Cambridge, Massachusetts; 5 Chinese Academy of Medical Sciences & Peking Union Medical College, Beijing, China; 6 Institute of Clinical Medical Sciences, China-Japan Friendship Hospital, Beijing, China; 7 Center for Evidence-Based Medicine, Capital Institute of Pediatrics, Beijing, China; 8 The Warren Alpert Medical School of Brown University, Providence, Rhode Island

## Abstract

**Importance:**

Obesity, especially visceral obesity, is an established risk factor associated with all-cause mortality. However, the inadequacy of conventional anthropometric measures in assessing fat distribution necessitates a more comprehensive indicator, body roundness index (BRI), to decipher its population-based characteristics and potential association with mortality risk.

**Objective:**

To evaluate the temporal trends of BRI among US noninstitutionalized civilian residents and explore its association with all-cause mortality.

**Design, Setting, and Participants:**

For this cohort study, information on a nationally representative cohort of 32 995 US adults (age ≥20 years) was extracted from the National Health and Nutrition Examination Survey (NHANES) from 1999 to 2018 and NHANES Linked Mortality File, with mortality ascertained through December 31, 2019. Data were analyzed between April 1 and September 30, 2023.

**Exposures:**

Biennial weighted percentage changes in BRI were calculated. Restricted cubic spline curve was used to determine optimal cutoff points for BRI.

**Main Outcome and Measures:**

The survival outcome was all-cause mortality. Mortality data were obtained from the Centers for Disease Control and Prevention website and linked to the NHANES database using the unique subject identifier. Weibull regression model was adopted to quantify the association between BRI and all-cause mortality.

**Results:**

Among 32 995 US adults, the mean (SD) age was 46.74 (16.92) years, and 16 529 (50.10%) were women. Mean BRI increased gradually from 4.80 (95% CI, 4.62-4.97) to 5.62 (95% CI, 5.37-5.86) from 1999 through 2018, with a biennial change of 0.95% (95% CI, 0.80%-1.09%; *P* < .001), and this increasing trend was more obvious among women, elderly individuals, and individuals who identified as Mexican American. After a median (IQR) follow-up of 9.98 (5.33-14.33) years, 3452 deaths (10.46% of participants) from all causes occurred. There was a U-shaped association between BRI and all-cause mortality, with the risk increased by 25% (hazard ratio, 1.25; 95% CI, 1.05-1.47) for adults with BRI less than 3.4 and by 49% (hazard ratio, 1.49; 95% CI, 1.31-1.70) for those with BRI of 6.9 or greater compared with the middle quintile of BRI of 4.5 to 5.5 after full adjustment.

**Conclusions and Relevance:**

This national cohort study found an increasing trend of BRI during nearly 20-year period among US adults, and importantly, a U-shaped association between BRI and all-cause mortality. These findings provide evidence for proposing BRI as a noninvasive screening tool for mortality risk estimation, an innovative concept that could be incorporated into public health practice pending consistent validation in other independent cohorts.

## Key Points

**Question:**

What are the temporal trends of body roundness index (BRI) and its association with all-cause mortality among US adults?

**Findings:**

In this cohort study involving 32 995 US adults, mean BRI increased from 4.80 to 5.62 during the period between 1999 and 2018, with a biennial change of 0.95%. The association between BRI and all-cause mortality followed a U-shape, with both lowest and highest BRI groups experiencing significantly increased risk of all-cause mortality.

**Meaning:**

These findings suggest that BRI may be promising as a newer anthropometric measure associated with all-cause mortality.

## Teaser

This cohort study evaluates temporal trends of body roundness index in a nationally representative sample of US adults and examines the associations of body roundness index and all-cause mortality.

## Introduction

Obesity is a global epidemic with high prevalence and contributes to increasing mortality rates. Globally, more than 1 billion people are obese.^1^ As 1 of the top 5 risk factors for mortality, obesity was associated with approximately 5 million deaths worldwide in 2019.^2^ Hence, a better understanding of the obesity-mortality association may optimize risk assessment, formulate antiobesity strategies, and prioritize rational planning of health care resources.

Generally, obesity is scaled by body mass index (BMI; calculated as weight in kilograms divided by height in meters squared). Numerous studies have demonstrated that obesity as defined by BMI was significantly associated with high risk of all-cause mortality compared with BMI within the reference range.^3^ With the extensive investigation of body composition, more attention has been paid to the association between visceral obesity and mortality.^4,5^ It is widely recognized that body fat content cannot be characterized by BMI. For instance, among individuals with the same BMI, fat distribution and body composition varied dramatically.^6^ A health check-up cohort study of 36 656 participants by Lee et al^7^ found that body fat distribution as reflected by visceral-to-subcutaneous fat area ratio was more consistently associated with of all-cause mortality than overall adiposity as reflected by BMI, after an mean follow-up of 5.7 years. Moreover, the prognostic capability of BMI outside reference range was found to be hinged on anthropometric and clinical conditions.^8,9^ To better embody fat distribution, a newer anthropometric measure, body roundness index (BRI), was coined by Thomas et al,^10^ who developed elliptical models based on human body shape to calculate body roundness and used eccentricity to estimate visceral fat and total body fat percentages. Besides weight and height, BRI additionally considers waist circumference, and hence it can more comprehensively reflect visceral fat distribution. BRI was found to be superior over other anthropometric indicators in estimating the risk for various clinical end points, including cardiometabolic disease,^11,12,13^ kidney disease,^14^ and cancer.^15^ Furthermore, longitudinal studies have shown that high BRI was associated with the significantly increased risk of all-cause mortality and cardiovascular disease-specific mortality.^16,17^ However, there is a paucity of national data on the association between BRI and mortality, and no study has been conducted among US general populations, to our knowledge. To fill this gap, we aimed to characterize the temporal trends of BRI among adults aged 20 years and older in a nationally representative US population sample from 1999 to 2018 and to examine the association between BRI and all-cause mortality.

## Methods

This cohort study was deemed exempt from ethical review and informed consent by the Capital Institute of Pediatrics academic review board owing to the use of deidentified, publicly available data. The National Health and Nutrition Examination Survey (NHANES) was approved by the National Center for Health Statistics Institutional Review Board, and all respondents provided written informed consent. This study follows the Strengthening the Reporting of Observational Studies in Epidemiology (STROBE) reporting guideline.

### Study Participants

All data have been made publicly available by the National Center for Health Statistics.^18^ Data from the NHANES, a series of nationally representative, cohort surveys designed to monitor public health of the US population, were used. Since 1999, NHANES has been conducted in 2-year cycles, collecting data from in-home interviews and study visits conducted at a mobile examination center.^19^ We included participants from 10 cycles of NHANES, from 1999 to 2018, who were nonpregnant adults aged 20 years or older and weighted to be representative of the noninstitutionalized civilian resident US population. The response rate of NHANES decreased from 76.62% for household interviews and 69.83% for medical examinations in 1999 to 2000 to 48.24% for household interviews and 45.70% for medical examinations in 2017 to 2018 (eTable 1 in Supplement 1).

### Sample Size

Of 59 064 respondents with complete eligibility status for mortality analysis, 6134 were excluded due to incomplete data on BRI, 1385 because they were pregnant, 11 518 owing to reporting a history of myocardial infarction, stroke, congestive heart failure, or cancer, and 7032 owing to missing information on covariates. The final sample size was 32 995 adults.

### Survival Outcome

The survival outcome was all-cause mortality. Mortality data were obtained from the Centers for Disease Control and Prevention website and linked to the NHANES database using the unique subject identifier, with death information ascertained through December 31, 2019.^20^ Causes of death were defined according to the *International Statistical Classification of Diseases and Related Health Problems, Tenth Revision* (*ICD-10*) codes. Study participants were followed up from the date of survey participation through the date of death or the end of the follow-up, whichever occurred first.

### BRI Definition

BRI was calculated as 364.2 − 365.5 × √(1 − [waist circumference in centimeters / 2π]^2^ / [0.5 × height in centimeters]^2^), according to the formula developed by Thomas et al.^10^ Waist circumference and body height were measured at mobile examination centers. Due to the lack of a reference range, BRI was categorized into 5 groups according to the 20th, 40th, 60th, and 80th quantiles to explore the association with all-cause mortality.

### Covariates

Data on age, sex, race and ethnicity, educational level, poverty income ratio (PIR; ratio of family income to poverty threshold, with a higher ratio representing a higher level of income.), smoking status, drinking status, family history of cardiovascular disease (CVD), and family history of diabetes were collected during in-home interviews. Race and ethnicity was self-reported by study participants based on questions with fixed category responses, including Mexican American, non-Hispanic Black, non-Hispanic White, and other race and ethnicity (American Indian or Alaska Native, Native Hawaiian or Pacific Islander, and non-Hispanic Asian). Race and ethnicity were included in analyses to test if BRI differs across these potentially confounding factors. Education was grouped as less than 9th grade, 9th to 11th grade, high school graduate, some college, and college graduate or above.

### Statistical Analysis

The complex survey design of NHANES was considered, and all results were weighted to provide nationally representative estimates for noninstitutionalized civilian US residents. Weighted proportions of study participants were calculated after combining data from 2 adjacent NHANES cycles (1999-2002, 2003-2006, 2007-2010, 2011-2014, and 2015-2018). Weighted mean BRIs and 95% CIs were calculated and compared across 10 cycles overall and in subgroups, with the trends examined by partial Mann-Kendall tests.

Restricted cubic spline (RCS) curve with 4 knots was displayed to test nonlinearity and determine optimal cutoff points for BRI when estimating all-cause mortality. Then, Cox model assumption of proportionality was checked for BRI and covariates. If this assumption was violated, Weibull proportional hazards assumption was tested, that is, the linear distribution between ln(−ln[S{t}]) and ln(t), where ln indicates natural log, S(t) denotes survival function, and t denotes survival time. The weighted association between BRI and all-cause mortality was quantified using hazard ratios (HRs) with 95% CIs before and after adjusting for confounding factors. First, only age and sex were adjusted. Second, age, sex, race and ethnicity, educational level, and PIR were adjusted. Third, age, sex, race and ethnicity, educational level, PIR, smoking status, drinking status, family history of CVD, and family history of diabetes were adjusted. Survival risk associated with BRI was also examined on stratification by these confounding factors on categorical scales.

Finally, to assess the robustness of association results, sensitivity analyses were performed by excluding adults with accidental deaths, by excluding adults who died within 2 years after participation, and by reserving participants reporting a history of myocardial infarction, stroke, congestive heart failure, or cancer, respectively.

All analyses were conducted using Stata software version 16.0 (StataCorp) and R programming environment version 3.5.2 (R Project for Statistical Computing). *P* values were 2-tailed, and *P* < .05 was considered statistically significant. Data were analyzed between April 1 and September 30, 2023.

## Results

### Baseline Characteristics

Among 32 995 eligible adults with complete data on BRI and mortality, the mean (SD) age was 46.74 (16.92) years, and 16 529 (50.10%) were women. After survey weighting, 8.53% (95% CI, 7.46%-9.74%) of participants were Mexican American, 10.92% (95% CI, 9.84%-12.10%) of participants were non-Hispanic Black, 68.26% (95% CI, 66.14%-70.31%) of participants were non-Hispanic White, and 12.29% (95% CI, 11.25%-13.41%) of participants identified as other race or ethnicity. The baseline characteristics of study participants are shown in Table 1 after combining every 2 consecutive cycles of NHANES data set.

**Table 1.** Baseline Characteristics of the US Adults in the National Health and Nutrition Examination Survey, 1999 to 2018^a^

Characteristics	Participants by survey wave											
	1999-2002 (n = 5860)		2003-2006 (n = 6036)		2007-2010 (n = 7422)		2011-2014 (n = 7042)			2015-2018 (n = 6635)		
	No.	% (95% CI)	No.	% (95% CI)	No.	% (95% CI)	No.	% (95% CI)	No.		% (95% CI)
Age, y^b^											
20 to <45	2839	55.62 (53.10-58.10)	2969	54.17 (51.27-57.04)	3541	52.47 (50.40-54.53)	3496	51.26 (48.58-53.93)	3096		50.02 (47.40-52.63)
45 to <65	1897	33.05 (30.76-35.43)	1925	34.66 (32.32-37.07)	2586	36.16 (34.46-37.89)	2461	36.88 (34.80-39.01)	2425		36.37 (34.36-38.42)
≥65	1124	11.33 (10.39-12.34)	1142	11.17 (9.82-12.68)	1295	11.37 (10.52-12.28)	1085	11.86 (10.65-13.19)	1114		13.62 (11.92-15.51)
Sex^b^											
Female	2880	49.83 (48.49-51.18)	2970	50.49 (49.47-51.52)	3768	50.63 (49.39-51.87)	3514	49.77 (48.15-51.40)	3397		50.66 (49.21-52.12)
Male	2980	50.17 (48.82-51.51)	3066	49.51 (48.48-50.53)	3654	49.37 (48.13-50.61)	3528	50.23 (48.60-51.85)	3238		49.34 (47.88-50.79)
Race and ethnicity^c^											
Mexican American	1471	7.35 (5.62-9.55)	1295	8.29 (6.21-11.00)	1403	8.87 (6.33-12.30)	844	8.69 (6.37-11.75)	1095		9.30 (6.78-12.64)
Non-Hispanic Black	1113	10.18 (8.02-12.84)	1332	11.19 (8.74-14.21)	1431	11.11 (9.05-13.57)	1637	11.28 (8.79-14.37)	1434		10.80 (8.33-13.91)
Non-Hispanic White	2779	71.20 (67.50-74.73)	2966	71.69 (66.91-76.03)	3471	69.25 (63.98-74.06)	2756	65.98 (60.46-71.09)	2192		63.82 (58.58-68.75)
Other	497	11.23 (8.06-15.43)	443	8.83 (7.25-10.71)	1117	10.77 (8.66-13.32)	1805	14.06 (12.10-16.26)	1914		16.07 (13.66-18.82)
Education^b^											
<9th Grade	869	5.81 (4.87-6.90)	720	5.38 (4.46-6.47)	812	5.35 (4.43-6.44)	466	3.94 (3.32-4.67)	576		3.87 (2.93-5.12)
9th-11th Grade	1008	13.31 (11.92-14.84)	853	10.30 (8.87-11.92)	1223	12.64 (11.27-14.14)	913	10.10 (8.28-12.27)	713		7.33 (6.23-8.60)
High school graduate	1370	25.83 (23.61-28.18)	1456	25.09 (23.65-26.58)	1774	24.02 (22.33-25.81)	1516	21.06 (19.00-23.27)	1527		24.38 (22.08-26.84)
Some college	1478	29.20 (27.50-30.97)	1756	32.15 (30.26-34.11)	2076	30.28 (28.89-31.71)	2244	33.21 (31.28-35.21)	2082		31.57 (29.36-33.87)
≥College graduate	1135	25.85 (22.60-29.40)	1251	27.08 (24.27-30.10)	1537	27.72 (25.21-30.37)	1903	31.68 (28.3-35.27)	1737		32.85 (28.42-37.61)
PIR^b^											
<1	1036	13.22 (11.75-14.85)	1041	11.22 (9.86-12.74)	1538	13.66 (12.10-15.38)	1595	15.88 (13.33-18.80)	1288		12.81 (11.19-14.62)
≥1	4824	86.78 (85.15-88.25)	4995	88.78 (87.26-90.14)	5884	86.34 (84.62-87.90)	5447	84.12 (81.20-86.67)	5347		87.19 (85.38-88.81)
Cigarette smoking											
Yes	2794	48.70 (45.66-51.76)	2836	47.50 (45.82-49.19)	3346	44.46 (42.02-46.92)	2941	42.20 (39.94-44.49)	2669		41.39 (38.98-43.85)
No	3066	51.30 (48.24-54.34)	3200	52.50 (50.81-54.18)	4076	55.54 (53.08-57.98)	4101	57.80 (55.51-60.06)	3966		58.61 (56.15-61.02)
Alcohol drinking											
Yes	4094	73.66 (69.47-77.46)	4282	74.81 (72.20-77.25)	5441	77.80 (75.85-79.64)	5244	80.27 (77.34-82.90)	5280		85.38 (83.71-86.91)
No	1766	26.34 (22.54-30.53)	1754	25.19 (22.75-27.80)	1981	22.20 (20.36-24.15)	1798	19.73 (17.10-22.66)	1355		14.62 (13.09-16.29)
Family history of CVD											
Yes	452	9.50 (8.61-10.48)	694	13.27 (12.01-14.64)	898	11.72 (10.69-12.82)	752	11.72 (10.39-13.20)	775		11.95 (11.05-12.92)
No	5408	90.50 (89.52-91.39)	5342	86.73 (85.36-87.99)	6524	88.28 (87.18-89.31)	6290	88.28 (86.80-89.61)	5860		88.05 (87.08-88.95)
Family history of diabetes											
Yes	2890	49.52 (47.08-51.97)	2830	45.11 (42.71-47.53)	2984	36.97 (35.48-38.49)	2802	36.24 (34.28-38.24)	3030		42.69 (40.61-44.79)
No	2970	50.48 (48.03-52.92)	3206	54.89 (52.47-57.29)	4438	63.03 (61.51-64.52)	4240	63.76 (61.76-65.72)	3605		57.31 (55.21-59.39)

Abbreviations: CVD, cardiovascular disease; PIR, poverty impact ratio.

^a^ Nationally representative estimates of the nonpregnant US population aged 20 years or older. Percentage estimates were nationally representative through the use of survey weights.

^b^ Age, sex, education, and PIR were based on self-report. Income was converted to the ratio of family income to poverty according to the Department of Health and Human Services poverty thresholds.

^c^ Race and ethnicity were based on self-report in closed categories, and other race and ethnicity included American Indian or Alaska Native, Native Hawaiian or Pacific Islander, and non-Hispanic Asian.

### Temporal Trends of BRI

The temporal trends of BRI are presented in Table 2. Mean BRI increased from 4.80 (95% CI, 4.62-4.97) to 5.62 (95% CI, 5.37-5.86), with a biennial change of 0.95% (95% CI, 0.80%-1.09%; *P* < .001 from 1999 through 2018. Using the mean BRI from the 1999 to 2000 cycle as a reference, the changes ranged from −0.05 to 0.82 and differed significantly starting in 2007. Overall temporal trends of BRI among US adults were statistically significant (eFigure 1 in Supplement 1).

**Table 2.** Magnitude of Changes in Mean BRI for Subsequent National Health and Nutrition Examination Survey Cycles^a^

Survey years	BRI	Difference	*P* value for difference in BRI^b^	Overall *P* value for trend^c^
	Mean (SE) [95% CI]			
1999-2000	4.80 (0.09) [4.62-4.97]	0 [Reference]	NA	<.001
2001-2002	4.74 (0.05) [4.64-4.85]	−0.05	.54	
2003-2004	4.98 (0.07) [4.84-5.13]	0.19	.17	
2005-2006	5.01 (0.09) [4.83-5.20]	0.22	.16	
2007-2008	5.11 (0.07) [4.98-5.24]	0.32	.01	
2009-2010	5.12 (0.06) [5.00-5.25]	0.33	.01	
2011-2012	5.25 (0.09) [5.08-5.42]	0.45	.002	
2013-2014	5.39 (0.07) [5.25-5.54]	0.60	<.001	
2015-2016	5.52 (0.11) [5.29-5.74]	0.72	<.001	
2017-2018	5.62 (0.12) [5.37-5.86]	0.82	<.001	

Abbreviations: BRI, body roundness index; NA, not applicable.

^a^ Nationally representative estimates of the nonpregnant US population aged 20 years or more. Estimates were nationally representative through the use of survey weights.

^b^ *P* value for difference in mean BRI was calculated using the linear combinations of parameters.

^c^ *P* value for overall trend was calculated using the Mann-Kendall trend test.

**Figure 1.**
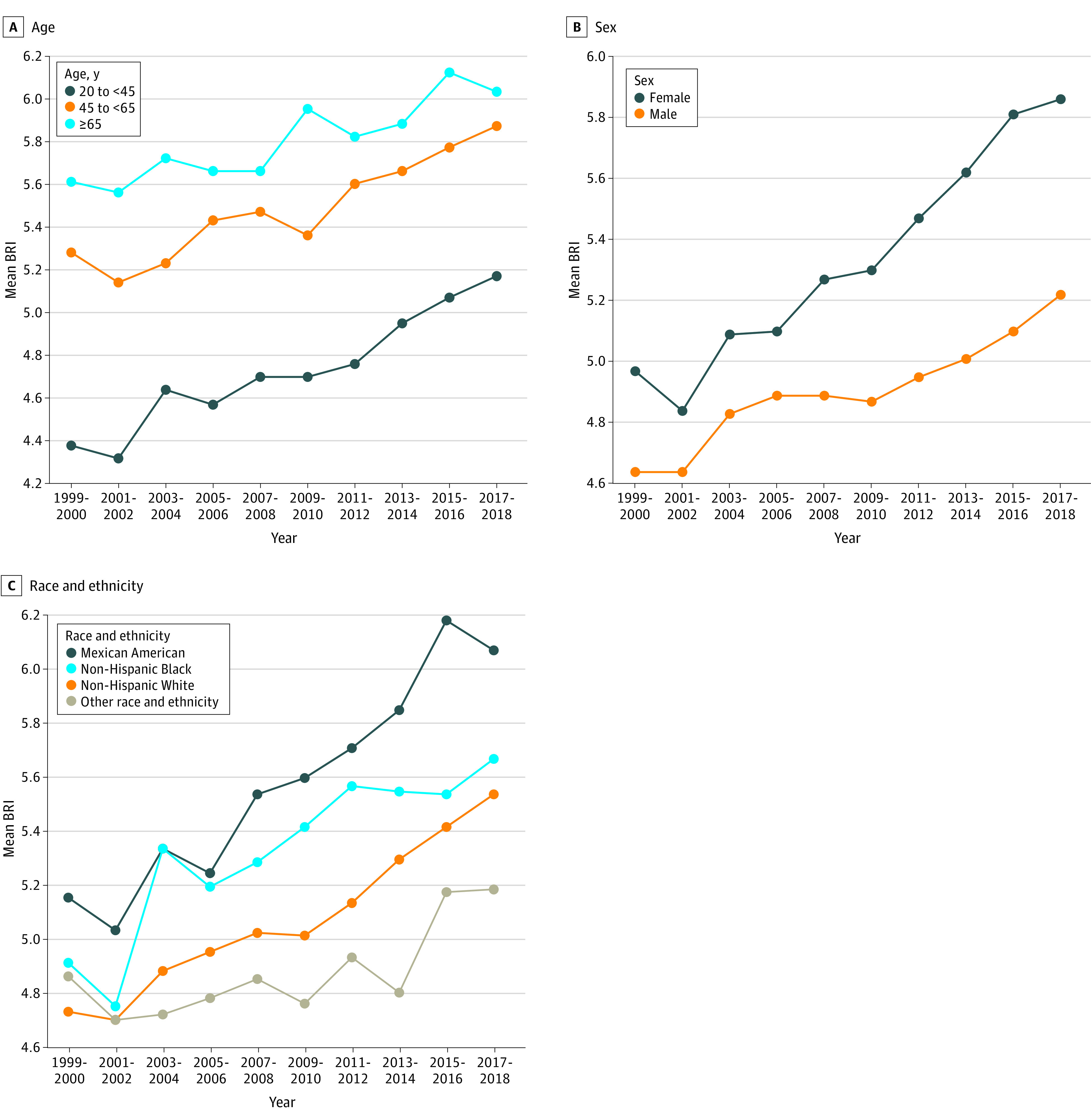
Trends of Mean BRI Values by Age, Sex, and Race and Ethnicity in US Adults Nationally representative estimates of the nonpregnant US population aged 20 years or older. Estimates were nationally representative through the use of survey weights from the National Health and Nutrition Examination Survey. Race and ethnicity were based on self-report in closed categories, and other race and ethnicity included American Indian or Alaska Native, and Native Hawaiian or Pacific Islander, and non-Hispanic Asian. BRI indicates body roundness index.

Additionally, BRI trends across NHANES cycles were summarized stratified by sociodemographic factors (Figure 1; eFigure 2 in Supplement 1). By age groups, BRI increased with aging and across cycles. Generally, BRI was higher in women than in men, and exhibited an increasing tendency, with the difference between sexes being gradually widened from 1999 through 2018. By race and ethnicity, BRI was highest among Mexican American participants, followed by non-Hispanic Black participants and non-Hispanic White participants. By education, adults with college graduate degree or above had the lowest BRI, and in a sharp contrast, the highest BRI was consistently seen for adults with education less than 9th grade. By family income, BRI was generally high among adults with PIR less than 1 compared with PIR 1 or greater, and divergence before and after the 2011 to 2012 cycle was more obvious. By cigarette smoking, mean BRIs were comparable before 2009, but increased significantly in smokers compared with nonsmokers afterwards. By alcohol drinking, except for the 2017 to 2018 cycle, nondrinkers had a higher BRI level than drinkers, and the time trends increased in parallel. Among adults with a family history of CVD or diabetes, BRI was consistently elevated compared with those without, and their increasing trends widened from 1999 through 2018. All subsidiary trends over nearly 2 decades remained statistically significant at α = .05 (eTable 2 in Supplement 1).

### BRI and All-Cause Mortality

**Figure 2.**
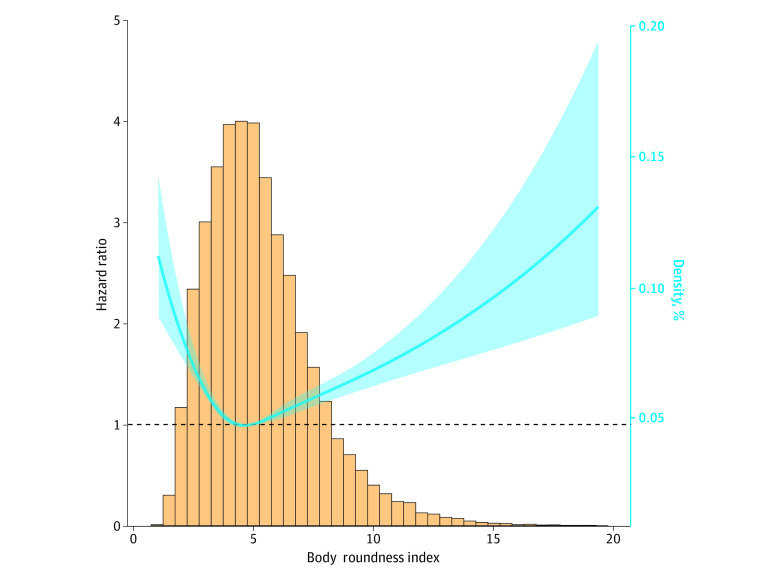
The Association Between Body Roundness Index and All-Cause Mortality Risk After Full Adjustment The solid curved line represents the estimates for the association of BRI with all-cause mortality, and shading, the 95% CI.

During a median (IQR) follow-up of 9.98 (5.33-14.33) years, 3452 deaths (10.46% of participants) occurred. Given no recommended cutoff points of BRI thus far, an RCS curve was first adopted to display its association with all-cause mortality (Figure 2). This association followed a U-shaped risk trajectory. Then, BRI was categorized in quintiles (Q1, lowest, to Q5, highest) and Q3 (4.45 to <5.46) was assigned as the reference group, consistent with the insignificant interval of the RCS curve.

Schoenfeld test found that the proportional hazards assumption was invalid. Viewing the linear association between ln(−ln[S{t}]) and ln(t) (eFigure 3 in Supplement 1), Weibull regression model was used for BRI to estimate all-cause mortality.

The associations of BRI in categories with mortality before and after adjusting for sociodemographic factors are provided in Table 3. The risk for all-cause mortality was statistically significant for Q1 (BRI, 1.05 to <3.41), Q4 (BRI, 5.46 to <6.91), and Q5 (BRI, ≥6.91) compared with Q3 after controlling varying panels of confounders. Specifically, after full adjustment, adults within Q1 were 25% more likely to die from any cause compared with adults within Q3 (hazard ratio [HR], 1.25; 95% CI, 1.05 to 1.47); findings were similar for adults in Q4 (HR, 1.25; 95% CI, 1.09 to 1.43), and adults in Q5 were 50% more likely to die from any cause (HR, 1.49; 95% CI, 1.31 to 1.70).

**Table 3.** Adjusted HR of All-Cause Mortality According to the Quantiles of Body Roundness Index^a^

Weibull Regression Model	HR (95% CI)				
	Q1: 1.05 to <3.41	Q2: 3.41 to <4.45	Q3: 4.45 to <5.46	Q4: 5.46 to <6.91	Q5: ≥6.91
Without adjustment	0.57 (0.49-0.67)^b^	0.81 (0.69-0.95)^c^	1 [Reference]	1.48 (1.30-1.69)^b^	1.62 (1.42-1.85)^b^
Adjusted only for age and sex	1.21 (1.03-1.44)^c^	1.10 (0.95-1.28)	1 [Reference]	1.31 (1.15-1.49)^b^	1.58 (1.39-1.81)^b^
Partially adjusted^d^	1.26 (1.07-1.49)^e^	1.13 (0.97-1.31)	1 [Reference]	1.26 (1.10-1.44)^b^	1.52 (1.33-1.74)^b^
Fully adjusted^f^	1.25 (1.05-1.47)^c^	1.13 (0.97-1.31)	1 [Reference]	1.25 (1.09-1.43)^e^	1.49 (1.31-1.70)^b^

Abbreviations: HR, hazard ratio; Q, quantile.

^a^ Data on US adults 20 years or older from the National Health and Nutrition Examination Survey Linked Mortality Files, 1999 to 2018. There were 3452 all-cause deaths in 32 995 participants (329 393 total person-years).

^b^ *P* < .001.

^c^ *P* < .05.

^d^ Adjusted for age, sex, race and ethnicity, education, and income.

^e^ *P* < .01.

^f^ Adjusted for age, sex, race and ethnicity, education, income, smoking, drinking, family history of cardiovascular disease, and family history of diabetes.

Subgroup analyses by sociodemographic factors for the comparison of Q1 and Q5 with Q3 in estimating all-cause mortality were conducted after full adjustment (eFigure 4 in Supplement 1). Compared with Q3, significant mortality risk of Q1 was observed in adults aged 45 years or older (age 45-65 years: HR, 1.41; 95% CI, 1.04 to 1.90; age ≥65 years: 1.51; 95% CI, 1.22 to 1.86), males (HR, 1.41; 95% CI, 1.13 to 1.77), those with PIR of 1 or greater (HR, 1.28; 95% CI, 1.06 to 1.54), smokers (HR, 1.26; 95% CI, 1.03 to 1.54), and those who drank alcohol (HR, 1.23; 95% CI, 1.01 to 1.50). By contrast, significance of Q5 was consistently observed except female participants, those with high school education or above, those with PIR less than 1, and those with a family history of CVD. Additionally, forest plots for the models adjusting for age and sex, as well as the partially adjusted model are shown in eFigure 5 in Supplement 1 for the comparison of Q1 with Q3, and in eFigure 6 in Supplement 1 for the comparison of Q5 with Q3.

### Sensitivity Analyses

Sensitivity analyses were performed to test the stability and extrapolation of association results. The association between BMI and all-cause mortality risk is shown in eFigure 7 and eTable 3 in Supplement 1. Compared with BMI, BRI had narrower CIs and higher sensitivity in estimating risk for all-cause mortality. Significant estimation remained after excluding accidental deaths (eTable 4 in Supplement 1) and deaths within 2 years after participation (eTable 5 in Supplement 1), even reserving participants reporting a history of myocardial infarction, stroke, congestive heart failure, or cancer (eTable 6 in Supplement 1).

## Discussion

The aim of this cohort study was to characterize the temporal trends of BRI among US adults aged at least 20 years from 1999 to 2018 and to explore the association of BRI with all-cause mortality. Of note, BRI experienced a stably increasing trend during nearly 2 decades, with a biennial change of 0.95%. This trend was more obvious among women, individuals aged 65 years or older, and Mexican American participants. Moreover, there was a U-shaped association between BRI and all-cause mortality, with the risk increased by 25% for those with BRI less than 3.4 and by 49% with BRI of 6.9 or greater compared with to the middle quantile of 4.5 to 5.5. To our knowledge, this is the first study that has evaluated the trends of BRI and its associations with all-cause mortality in US general populations.

Obesity, particularly visceral obesity, is recognized as an established risk factor associated with cardiovascular events and all-cause mortality. Currently, there is a growing consensus that visceral fat is much more dangerous to health than subcutaneous fat, since it entails more risk for diseases.^21,22^ In support of this notion, a study by Kuk et al^23^ reported that compared with subcutaneous and liver fat, visceral fat determined by computed tomography was a significant, independent risk factor associated with all-cause mortality. For practical reasons, there remains a need for a simple and effective proxy indicator to better reflect visceral obesity. Accruing evidence indicates that BRI, as a newer anthropometric measure, can reflect visceral fat more comprehensively than conventional measures, including BMI. Theoretically, assuming the shape of body as an ellipse with the long axis height and the short axis waist circumference, BRI can be calculated as the eccentricity of this ellipse via human modeling. It is hence reasonable to speculate BRI as a superior anthropometric measure for abdominal adiposity.

Although the idea that BRI can estimate the percentages of total and regional fat may be plausible and appealing, evidence on the association between BRI and a disease or mortality is sparse. A study by Wu et al^17^ found that the risk for incident cardiovascular events increased with BRI in a dose-dependent manner among 59 278 participants free of malignant tumors and cardiovascular diseases, especially in younger adults. A study by Liu et al^24^ followed up 6990 hypertensive adults without diabetes for 3 years and observed that BRI was superior over the other anthropometric measures in estimating the onset of diabetes.^24^ A study by Zhou et al^25^ reported that high BRI quartiles were associated with significantly reducing risk of all-cause mortality by 17% to 27% and of cardiovascular mortality by 21% to 22% among 47 356 adults from NHANES from 1999 to 2014. As an extension, we expanded NHANES cycles from 1999 through 2018 and followed up to December 31, 2019, and we observed a U-shaped risk trajectory for the association between BRI and all-cause mortality. Differing from the study by Zhou et al,^25^ we chose the middle quantile (BRI, 4.5-5.5) of this U-shaped trajectory as the reference and found that all-cause mortality risk was increased by 25% for adults with BRI less than 3.4 and by nearly 50% for adults with BRI 6.9 or greater. The magnitude of risk estimation persisted even after excluding accidental deaths or deaths within 2 years or reserving participants with myocardial infarction, stroke, congestive heart failure, or cancer. Hence, estimates of mortality risk associated with BRI may help inform decision-making in clinical settings.

In this national cohort, we noticed that very low BRI was associated with a significantly increased risk of all-cause mortality, especially in individuals aged 65 years and older. This association seems plausible, as BRI was identified as a potential proxy measure associated with nutritional status,^26^ and very low BRI can be accompanied with malnutrition, fatigue, reduced activity tolerance, and muscle atrophy.^25^ The reasons behind the association between BRI and mortality may be epidemiologically and clinically plausible. From epidemiological aspects, elevated BRI was significantly associated with an increased risk of cardiovascular and metabolic disorders, and even cancer,^11,15,27,28,29^ which might serve as the culprits responsible for all-cause mortality. From clinical aspects, the accumulation of visceral fat was associated with more profound insulin resistance and an increased risk of cardiometabolic diseases, even among participants with weight within reference range.^30,31^

It is also worth noting that in this study, BRI exhibited an overall upward trend from 1999 to 2018, in parallel to the prevalence of US obesity and central adiposity,^32^ with this increasing trend reaching statistical significance in cycle-to-cycle comparisons starting in 2007. Alarmingly, the overall mean BRI exceeded the upper limit of the bottom range of the U-shaped risk trajectory in this study after 2015. Moreover, our subsidiary observations indicated that the increasing trends in BRI might be more obvious among women, individuals older than 65 years, and Mexican American individuals, which could enhance our understanding on BRI distributions to inform programs or guidance for body shape management from the following 3 aspects. First, the impact of sex hormones on body composition and appetite may explain the relatively high BRI in women.^33^ Second, high BRI in individuals older than 65 years may be indicative of adipose tissue senescence and dysfunction.^34,35^ Third, poor dietary quality, food insecurity, and psychosocial stress are not uncommon among Mexican American individuals,^36^ which might explain the high BRI.

### Strengths and Limitations

Besides the strengths of this study, including comprehensive analyses of nationally representative samples, long-term follow-ups, and careful considerations of multiple confounding factors, some limitations should be acknowledged. First, from 1999 through 2018, the response rates of NHANES declined from 76.62% to 48.24%, leaving the possibility of nonresponse bias an open question. Second, only all-cause mortality was evaluated, and disease-specific mortality remained unexplored due to small sample sizes. Third, the bottom range of BRI in the U-shaped risk trajectory with all-cause mortality (ie, Q3) was derived from general US populations, and it might differ across races and ethnicities because visceral adiposity deposits have been reported to be the highest in Hispanic individuals (eg, Mexican American individuals), followed by non-Hispanic Black individuals and non-Hispanic White individuals.^37^

## Conclusions

In this national cohort study, our findings indicated an increasing trend of BRI during nearly 20-year period among US adults, and importantly, a U-shaped association between BRI and all-cause mortality. Our findings provide compelling evidence for the application of BRI as a noninvasive and easy to obtain screening tool for estimation of mortality risk and identification of high-risk individuals, a novel concept that could be incorporated into public health practice pending consistent validation in other independent studies.

## Notes

Supplement 1. **eTable 1.** Overall Unweighted Response Rate for Household Interview and Medical Examination, NHANES 1999-2000 to 2017-2018 **eTable 2.** Estimated Mean Values of BRI by Sociodemographic Factors in US Adults From the NHANES, 1999 to 2018 **eTable 3.** Adjusted HR of All-Cause Mortality According to the Quintiles of BMI **eTable 4.** Adjusted HR of All-Cause Mortality According to the Quintiles of BRI After Excluding Accidental Deaths **eTable 5.** Adjusted HR of All-Cause Mortality According to the Quintiles of BRI After Excluding Deaths Within 2 Years After Survey **eTable 6.** Adjusted HR of All-Cause Mortality According to the Quintiles of BRI After Reserving Those Reporting a History of Myocardial Infarction, Stroke, Congestive Heart Failure, or Cancer **eFigure 1.** Trends of Mean BRI Values in US Adults From the NHANES, 1999 to 2018 **eFigure 2.** Trends of BRI by Socioeconomic Factors in US Adults From 1999 to 2018 **eFigure 3.** The Distribution Plot of ln(-ln(S(t))) Relative to ln(t) for Assessing Cox Assumption of Body Roundness Index in Quintiles **eFigure 4.** Adjusted HR of All-Cause Mortality by Sociodemographic Factors Comparing Quintile 1 and Quintile 5 With Quintile 3 of BRI in US Adults From the NHANES Linked Mortality Files, 1999 to 2018, in Fully Adjusted Model **eFigure 5.** Adjusted HR of All-Cause Mortality by Sociodemographic Factors Comparing Quintile 1 With Quintile 3 of BRI in US Adults From the NHANES Linked Mortality Files, 1999 to 2018, After Adjusting For Only Age and Sex and in Partially Adjusted Model **eFigure 6.** Adjusted HR of All-Cause Mortality by Sociodemographic Factors Comparing Quintile 1 With Quintile 3 of BRI in US Adults From the NHANES Linked Mortality Files, 1999 to 2018, After Adjusting for Only Age and Sex and in Partially Adjusted Model **eFigure 7.** The Association Between Body Round Index and All-Cause Mortality Risk After Full Adjustment

Supplement 2.
Data Sharing Statement
