# Error in Formula

**DOI:** 10.1001/jamanetworkopen.2024.26540

**Published:** 2024-07-11

In the Original Investigation titled “Body Roundness Index and All-Cause Mortality Among US Adults,”^1^ published June 5, 2024, there was an error in the formula to calculate body roundness index. Height and waist circumference should be inserted into the formula using the same unit (eg, centimeters). This article has been corrected.^1^
