# Supplementary material for: Body Roundness Index and All-Cause Mortality Among US Adults
Source: JAMA Netw Open. 2024 Jun 5;7(6):e2415051. doi: 10.1001/jamanetworkopen.2024.15051 (PMC11154161; doi:10.1001/jamanetworkopen.2024.15051)
Supplement: Supplement 1. — eTable 1. Overall Unweighted Response Rate for Household Interview and Medical Examination, NHANES 1999-2000 to 2017-2018 eTable 2. Estimated Mean Values of BRI by Sociodemographic Factors in US Adults From the NHANES, 1999 to 2018 eTable 3. Adjusted HR of All-Cause Mortality According to the Quintiles of BMI eTable 4. Adjusted HR of All-Cause Mortality According to the Quintiles of BRI After Excluding Accidental Deaths eTable 5. Adjusted HR of All-Cause Mortality According to the Quintiles of BRI After Excluding Deaths Within 2 Years After Survey eTable 6. Adjusted HR of All-Cause Mortality According to the Quintiles of BRI After Reserving Those Reporting a History of Myocardial Infarction, Stroke, Congestive Heart Failure, or Cancer eFigure 1. Trends of Mean BRI Values in US Adults From the NHANES, 1999 to 2018 eFigure 2. Trends of BRI by Socioeconomic Factors in US Adults From 1999 to 2018 eFigure 3. The Distribution Plot of ln(-ln(S(t))) Relative to ln(t) for Assessing Cox Assumption of Body Roundness Index in Quintiles eFigure 4. Adjusted HR of All-Cause Mortality by Sociodemographic Factors Comparing Quintile 1 and Quintile 5 With Quintile 3 of BRI in US Adults From the NHANES Linked Mortality Files, 1999 to 2018, in Fully Adjusted Model eFigure 5. Adjusted HR of All-Cause Mortality by Sociodemographic Factors Comparing Quintile 1 With Quintile 3 of BRI in US Adults From the NHANES Linked Mortality Files, 1999 to 2018, After Adjusting For Only Age and Sex and in Partially Adjusted Model eFigure 6. Adjusted HR of All-Cause Mortality by Sociodemographic Factors Comparing Quintile 1 With Quintile 3 of BRI in US Adults From the NHANES Linked Mortality Files, 1999 to 2018, After Adjusting for Only Age and Sex and in Partially Adjusted Model eFigure 7. The Association Between Body Round Index and All-Cause Mortality Risk After Full Adjustment [file jamanetwopen-e2415051-s001.pdf]

Supplemental Online Content

Zhang X, Ma N, Lin Q, et al. Body roundness index and all-cause mortality among US adults. *JAMA Netw Open*. 2024;7(6):e2415051.
doi:10.1001/jamanetworkopen.2024.15051

eTable 1. Overall Unweighted Response Rate for Household Interview and Medical Examination, NHANES 1999-2000 to 2017-2018
eTable 2. Estimated Mean Values of BRI by Sociodemographic Factors in US Adults From the NHANES, 1999 to 2018
eTable 3. Adjusted HR of All-Cause Mortality According to the Quintiles of BMI
eTable 4. Adjusted HR of All-Cause Mortality According to the Quintiles of BRI After Excluding Accidental Deaths
eTable 5. Adjusted HR of All-Cause Mortality According to the Quintiles of BRI After Excluding Deaths Within 2 Years After Survey
eTable 6. Adjusted HR of All-Cause Mortality According to the Quintiles of BRI After Reserving Those Reporting a History of Myocardial Infarction, Stroke, Congestive Heart Failure, or Cancer
eFigure 1. Trends of Mean BRI Values in US Adults From the NHANES, 1999 to 2018
eFigure 2. Trends of BRI by Socioeconomic Factors in US Adults From 1999 to 2018.
eFigure 3. The Distribution Plot of ln(-ln(S(t))) Relative to ln(t) for Assessing Cox Assumption of Body Roundness Index in Quintiles
eFigure 4. Adjusted HR of All-Cause Mortality by Sociodemographic Factors Comparing Quintile 1 and Quintile 5 with Quintile 3 of BRI in US Adults From the NHANES Linked Mortality Files, 1999 to 2018, in Fully Adjusted Model
eFigure 5. Adjusted HR of All-Cause Mortality by Sociodemographic Factors Comparing Quintile 1 With Quintile 3 of BRI in US Adults From the NHANES Linked Mortality Files, 1999 to 2018, After Adjusting For Only Age and Sex and in Partially Adjusted Model
eFigure 6. Adjusted HR of All-Cause Mortality by Sociodemographic Factors Comparing Quintile 1 With Quintile 3 of BRI in US Adults From the NHANES Linked Mortality Files, 1999 to 2018, After Adjusting for Only Age and Sex and in Partially Adjusted Model
eFigure 7. The Association Between Body Round Index and All-Cause Mortality Risk After Full Adjustment

This supplemental material has been provided by the authors to give readers additional information about their work.

© 2024 Zhang X et al. *JAMA Network Open.*

eTable 1. Overall unweighted response rate for household interview and medical examination, NHANES 1999-2000 to 2017-2018 (2-year cycles).

Survey years	Unweighted response rate for household interview	Unweighted response rate for medical exam
1999-2000	76.62%	69.83%
2001-2002	69.83%	73.40%
2003-2004	72.87%	69.06%
2005-2006	74.44%	71.46%
2007-2008	73.76%	71.11%
2009-2010	73.76%	71.93%
2011-2012	67.97%	65.22%
2013-2014	66.35%	64.30%
2015-2016	64.30%	64.30%
2017-2018	48.24%	45.70%

Abbreviations: NHANES, National Health and Nutrition Examination Survey.

© 2024 Zhang X et al. *JAMA Network Open.*

eTable 2. Estimated Mean Values of BRI by Sociodemographic Factors in US Adults From the NHANES, 1999 to 2018.[a]

Subgroups	BRI, weighted mean (95% CI)										*P* for trend[b]
	1999-2000 (n = 2722)	2001-2002 (n = 3138)	2003-2004 (n = 2954)	2005-2006 (n = 3082)	2007-2008 (n = 3653)	2009-2010 (n = 3769)	2011-2012 (n = 3389)	2013-2014 (n = 3653)	2015-2016 (n = 3407)	2017-2018 (n = 3228)	
Age, yrs											
20-45	4.38 (4.18, 4.59)	4.32 (4.19, 4.45)	4.64 (4.49, 4.80)	4.57 (4.34, 4.80)	4.70 (4.53, 4.88)	4.70 (4.52, 4.87)	4.76 (4.53, 5.00)	4.95 (4.74, 5.15)	5.07 (4.84, 5.31)	5.17 (4.88, 5.46)	<0.001
45-65	5.28 (4.90, 5.66)	5.14 (4.93, 5.36)	5.23 (5.02, 5.45)	5.43 (5.17, 5.68)	5.47 (5.31, 5.63)	5.36 (5.18, 5.55)	5.60 (5.28, 5.91)	5.66 (5.47, 5.85)	5.77 (5.47, 6.07)	5.87 (5.55, 6.18)	0.001
≥65	5.61 (5.41, 5.81)	5.56 (5.43, 5.70)	5.72 (5.56, 5.88)	5.66 (5.50, 5.82)	5.66 (5.42, 5.89)	5.95 (5.70, 6.21)	5.82 (5.52, 6.12)	5.88 (5.58, 6.18)	6.12 (5.84, 6.41)	6.03 (5.81, 6.25)	0.005
Sex											
Female	4.97 (4.70, 5.24)	4.84 (4.68, 5.00)	5.09 (4.91, 5.27)	5.10 (4.85, 5.35)	5.27 (5.10, 5.44)	5.30 (5.18, 5.41)	5.47 (5.27, 5.67)	5.62 (5.39, 5.85)	5.81 (5.54, 6.09)	5.86 (5.48, 6.23)	<0.001
Male	4.64 (4.44, 4.84)	4.64 (4.55, 4.74)	4.83 (4.69, 4.96)	4.89 (4.68, 5.10)	4.89 (4.78, 5.01)	4.87 (4.68, 5.07)	4.95 (4.73, 5.16)	5.01 (4.91, 5.10)	5.10 (4.85, 5.36)	5.22 (5.05, 5.40)	<0.001
Race and Ethnicity											
Mexican American	5.16 (4.98, 5.33)	5.04 (3.43, 6.65)	5.34 (4.16, 6.52)	5.25 (4.52, 5.99)	5.54 (5.46, 5.62)	5.6 (5.03, 6.16)	5.71 (5.11, 6.31)	5.85 (2.98, 8.72)	6.18 (6.00, 6.35)	6.07 (5.05, 7.08)	<0.001
Non-Hispanic White	4.74 (4.28, 5.20)	4.71 (4.60, 4.82)	4.89 (3.25, 6.52)	4.96 (2.68, 7.23)	5.03 (4.45, 5.61)	5.02 (4.73, 5.30)	5.14 (4.84, 5.44)	5.30 (3.80, 6.80)	5.42 (2.46, 8.37)	5.54 (5.52, 5.56)	<0.001
Non-Hispanic Black	4.92 (4.75, 5.09)	4.76 (1.75, 7.77)	5.34 (4.97, 5.70)	5.20 (3.73, 6.67)	5.29 (2.73, 7.86)	5.42 (5.21, 5.63)	5.57 (5.17, 5.98)	5.55 (5.27, 5.83)	5.54 (4.34, 6.73)	5.67 (4.91, 6.43)	0.004
Other race[c]	4.87 (3.95, 5.79)	4.71 (3.52, 5.91)	4.73 (1.74, 7.72)	4.79 (3.47, 6.11)	4.86 (3.87, 5.85)	4.77 (4.64, 4.90)	4.94 (4.82, 5.06)	4.81 (4.80, 4.83)	5.18 (2.01, 8.34)	5.19 (5.06, 5.32)	0.03
Education											
Less than 9th grade	5.22 (4.56, 5.89)	5.38 (2.47, 8.30)	5.38 (4.43, 6.33)	5.39 (4.83, 5.95)	5.59 (0.83, 10.35)	5.74 (5.32, 6.16)	5.87 (5.53, 6.21)	5.88 (4.36, 7.39)	6.00 (4.40, 7.60)	5.96 (4.65, 7.28)	<0.001
9-11th grade	5.29 (4.5, 6.09)	4.83 (2.83, 6.82)	5.22 (2.11, 8.34)	5.26 (2.74, 7.77)	5.33 (3.64, 7.01)	5.48 (5.31, 5.65)	5.55 (4.80, 6.31)	5.52 (3.02, 8.03)	5.81 (5.77, 5.86)	5.56 (0.43, 10.69)	0.002

© 2024 Zhang X et al. *JAMA Network Open.*

High school graduate	5.01 (5.00, 5.02)	4.88 (4.87, 4.90)	5.27 (4.13, 6.41)	5.25 (2.74, 7.76)	5.34 (4.05, 6.64)	5.29 (5.07, 5.51)	5.63 (5.53, 5.73)	5.53 (3.47, 7.58)	5.68 (2.58, 8.78)	5.85 (3.81, 7.89)	0.001
Some college	4.77 (4.46, 5.09)	4.76 (4.16, 5.36)	4.95 (4.48, 5.42)	5.03 (4.51, 5.56)	5.08 (4.99, 5.17)	5.12 (4.97, 5.26)	5.23 (4.78, 5.69)	5.60 (5.00, 6.20)	5.66 (4.56, 6.76)	5.74 (5.71, 5.78)	<0.001
College graduate or above	4.20 (3.85, 4.56)	4.42 (3.89, 4.95)	4.49 (2.65, 6.33)	4.55 (2.05, 7.04)	4.62 (3.46, 5.79)	4.61 (4.45, 4.77)	4.70 (4.61, 4.80)	4.72 (4.56, 4.88)	4.98 (1.73, 8.22)	5.05 (3.28, 6.81)	<0.001
Poverty impact ratio											
<1	5.10 (4.84, 5.35)	4.92 (4.66, 5.19)	5.19 (4.81, 5.58)	5.11 (4.79, 5.42)	5.21 (4.91, 5.51)	5.22 (4.92, 5.51)	5.22 (4.82, 5.63)	5.50 (5.17, 5.82)	5.78 (5.48, 6.08)	5.77 (5.27, 6.27)	<0.001
≥1	4.76 (4.55, 4.97)	4.72 (4.62, 4.81)	4.93 (4.81, 5.05)	4.98 (4.77, 5.19)	5.07 (4.95, 5.19)	5.06 (4.93, 5.20)	5.20 (4.99, 5.41)	5.28 (5.13, 5.43)	5.42 (5.17, 5.66)	5.51 (5.29, 5.73)	<0.001
Cigarette smoking											
Yes	4.86 (4.63, 5.09)	4.74 (4.65, 4.84)	4.98 (4.81, 5.15)	4.93 (4.67, 5.20)	5.09 (4.98, 5.19)	5.08 (4.94, 5.21)	5.26 (5.09, 5.42)	5.41 (5.24, 5.58)	5.64 (5.32, 5.95)	5.72 (5.43, 6.01)	<0.001
No	4.75 (4.49, 5.01)	4.74 (4.58, 4.89)	4.95 (4.73, 5.16)	5.05 (4.84, 5.26)	5.08 (4.90, 5.27)	5.09 (4.90, 5.28)	5.17 (4.93, 5.41)	5.24 (4.99, 5.50)	5.34 (5.10, 5.58)	5.42 (5.21, 5.63)	<0.001
Alcohol drinking											
Yes	4.67 (4.44, 4.90)	4.56 (4.48, 4.65)	4.80 (4.66, 4.93)	4.85 (4.61, 5.08)	4.93 (4.79, 5.06)	4.95 (4.82, 5.08)	5.10 (4.89, 5.31)	5.21 (5.06, 5.36)	5.34 (5.09, 5.6)	5.55 (5.32, 5.78)	<0.001
No	5.17 (4.92, 5.42)	5.24 (4.94, 5.55)	5.43 (5.18, 5.67)	5.45 (5.13, 5.76)	5.59 (5.41, 5.77)	5.63 (5.43, 5.82)	5.67 (5.49, 5.84)	5.68 (5.35, 6.02)	5.87 (5.60, 6.14)	5.47 (4.88, 6.06)	0.002
Family history of CVD											
Yes	4.86 (4.74, 4.98)	5.01 (3.70, 6.32)	5.24 (5.12, 5.35)	5.36 (4.98, 5.73)	5.47 (5.01, 5.93)	5.59 (5.22, 5.96)	5.76 (5.38, 6.15)	5.78 (3.14, 8.41)	6.29 (3.44, 9.13)	6.25 (4.38, 8.13)	<0.001
No	4.80 (4.51, 5.09)	4.71 (4.45, 4.97)	4.93 (4.37, 5.49)	4.93 (2.69, 7.17)	5.03 (4.91, 5.16)	5.02 (4.99, 5.06)	5.13 (5.11, 5.15)	5.25 (4.78, 5.72)	5.35 (2.67, 8.03)	5.45 (5.42, 5.47)	<0.001
Family history of diabetes											
Yes	5.09 (4.78, 5.41)	4.97 (4.83, 5.12)	5.22 (5.05, 5.40)	5.46 (5.19, 5.74)	5.58 (5.43, 5.74)	5.49 (5.36, 5.62)	5.76 (5.55, 5.96)	5.89 (5.61, 6.16)	6.08 (5.82, 6.34)	5.92 (5.56, 6.27)	<0.001

© 2024 Zhang X et al. *JAMA Network Open.*

No	4.53 (4.32, 4.75)	4.51 (4.40, 4.61)	4.69 (4.53, 4.85)	4.68 (4.52, 4.85)	4.78 (4.61, 4.96)	4.86 (4.68, 5.03)	4.91 (4.71, 5.11)	4.97 (4.84, 5.10)	5.04 (4.82, 5.26)	5.25 (5.05, 5.44)	<0.001

Abbreviations: NHANES, National Health and Nutrition Examination Survey; BRI, body roundness index; 95% CI, 95% confidence interval; CVD: cardiovascular disease.

[a] Nationally representative estimates of the nonpregnant US population aged 20 years or more. Estimates were nationally representative through the use of survey weights.

[b] P value for trend was calculated using the Mann-Kendall trend test within each subgroup.

[c] Other race included non-Hispanic Asian, American Indian or Alaska Native, and Native Hawaiian or Pacifc Islander.

© 2024 Zhang X et al. *JAMA Network Open.*

eTable 3. Adjusted HR of All-Cause Mortality According to the Quintiles (Q1 to Q5) of BMI.[a]

Weibull Regression Model	HR (95% CI)				
	Q1	Q2	Q3	Q4	Q5
Range (13.18 to 82.10)	13.18 to <23.40	23.40 to <26.39	26.40 to <29.30	29.31 to <33.50	≥33.51
Without adjustment	0.92 (0.80, 1.10)	0.91 (0.79, 1.04)	1 [Ref]	1.10 (0.95, 1.27)	1.10 (0.93, 1.22)
Adjusted only for age and sex	1.46 (1.28, 1.66)***	1.05 (0.92, 1.19)	1 [Ref]	1.16 (1.02, 1.33)*	1.34 (1.17, 1.53)***
Partially adjusted[b]	1.47 (1.29, 1.68)***	1.07 (0.94, 1.21)	1 [Ref]	1.15 (1.00, 1.31)*	1.33 (1.16, 1.53)***
Fully adjusted[c]	1.44 (1.26, 1.64)***	1.06 (0.93, 1.20)	1 [Ref]	1.14 (0.99, 1.30)	1.31 (1.14, 1.51)***

Abbreviations: HR, hazard ratio; 95% CI, 95% confidence interval; BMI, body mass index; Ref, reference.

[a] Data on US adults 20 years or older from the National Health and Nutrition Examination Survey Linked Mortality Files, 1999 to 2018. There were 3452 all-cause deaths in 32995 participants (329393 total person-years).

[b] Adjusted for age, sex, race/ethnicity, education, and income.

[c] Adjusted for age, sex, race/ethnicity, education, income, smoking, drinking, family history of cardiovascular disease, and family history of diabetes.

*P < 0.05, **P < 0.01, ***P < 0.001.

© 2024 Zhang X et al. *JAMA Network Open.*

eTable 4. Adjusted HR of All-Cause Mortality According to the Quintiles (Q1 to Q5) of BRI After Excluding Accidental Deaths.[a]

Weibull Regression Model	HR (95% CI)				
	Q1	Q2	Q3	Q4	Q5
Without adjustment	0.56 (0.47, 0.67)***	0.81 (0.69, 0.96)*	1 [Ref]	1.48 (1.29, 1.70)***	1.66 (1.44, 1.92)***
Adjusted only for age and sex	1.22 (1.03, 1.47)*	1.12 (0.96, 1.30)	1 [Ref]	1.30 (1.13, 1.50)***	1.61 (1.40, 1.88)***
Partially adjusted[b]	1.25 (1.04, 1.49)*	1.13 (0.98, 1.32)	1 [Ref]	1.26 (1.09, 1.46)**	1.56 (1.35, 1.81)***
Fully adjusted[c]	1.23 (1.03, 1.47)*	1.13 (0.97, 1.32)	1 [Ref]	1.25 (1.08, 1.44)**	1.53 (1.33, 1.77)***

Abbreviations: HR, hazard ratio; 95% CI, 95% confidence interval; BRI, body roundness index; Ref, reference.

[a] Data on US adults 20 years or older from the National Health and Nutrition Examination Survey Linked Mortality Files, 1999 to 2018. After excluding accidental deaths, there were 3317 all-cause deaths in 32860 participants (328285 total person-years).

[b] Adjusted for age, sex, race/ethnicity, education and income.

[c] Adjusted for age, sex, race/ethnicity, education, income, smoking, drinking, family history of cardiovascular disease and family history of diabetes.

*P < 0.05, **P < 0.01, ***P < 0.001.

© 2024 Zhang X et al. *JAMA Network Open.*

eTable 5. Adjusted HR of All-Cause Mortality According to the Quintiles (Q1 to Q5) of BRI After Excluding Deaths within 2 years after Survey.[a]

Weibull Regression Model	HR (95% CI)				
	Q1	Q2	Q3	Q4	Q5
Without adjustment	0.55 (0.47, 0.66)***	0.81 (0.68, 0.95)*	1 [Ref]	1.47 (1.29, 1.69)***	1.64 (1.43, 1.88)***
Adjusted only for age and sex	1.20 (1.01, 1.43)*	1.11 (0.94, 1.29)	1 [Ref]	1.30 (1.12, 1.50)***	1.59 (1.39, 1.84)***
Partially adjusted[b]	1.25 (1.05, 1.49)*	1.13 (0.97, 1.33)	1 [Ref]	1.24 (1.07, 1.44)**	1.53 (1.32, 1.76)***
Fully adjusted[c]	1.23 (1.03, 1.47)*	1.13 (0.97, 1.32)	1 [Ref]	1.23 (1.06, 1.42)**	1.50 (1.31, 1.73)***

Abbreviations: HR, hazard ratio; 95% CI, 95% confidence interval; BRI, body roundness index; Ref, reference.

[a] Data on US adults 20 years or older from the National Health and Nutrition Examination Survey Linked Mortality Files, 1999 to 2018. After excluding deaths within 2 years after survey, there were 3107 all-cause deaths in 32650 participants (329002 total person-years).

[b] Adjusted for age, sex, race/ethnicity, education and income.

[c] Adjusted for age, sex, race/ethnicity, education, income, smoking, drinking, family history of cardiovascular disease and family history of diabetes.

*P < 0.05, **P < 0.01, ***P < 0.001.

© 2024 Zhang X et al. *JAMA Network Open.*

eTable 6. Adjusted HR of All-Cause Mortality According to the Quintiles (Q1 to Q5) of BRI After reserving those reporting a history of myocardial infarction, stroke, congestive heart failure, or cancer.[a]

Weibull Regression Model	HR (95% CI)				
	Q1	Q2	Q3	Q4	Q5
Without adjustment	0.53 (0.47, 0.60)***	0.76 (0.67, 0.86)***	1 [Ref]	1.38 (1.25, 1.53)***	1.53 (1.38, 1.70)***
Adjusted only for age and sex	1.19 (1.05, 1.34)***	1.02 (0.91, 1.15)	1 [Ref]	1.26 (1.14, 1.40)***	1.50 (1.34, 1.66)***
Partially adjusted[b]	1.24 (1.10, 1.40)***	1.06 (0.94, 1.18)	1 [Ref]	1.22 (1.10, 1.36)***	1.43 (1.29, 1.60)***
Fully adjusted[c]	1.22 (1.09, 1.37)***	1.07 (0.95, 1.20)	1 [Ref]	1.21 (1.09, 1.35)***	1.40 (1.26, 1.56)***

Abbreviations: HR, hazard ratio; 95% CI, 95% confidence interval; BRI, body roundness index; Ref, reference.

[a] Data on US adults 20 years or older from the National Health and Nutrition Examination Survey Linked Mortality Files, 1999 to 2018. After excluding accidental deaths, there were 5905 all-cause deaths in 39509 participants (380787 total person-years).

[b] Adjusted for age, sex, race/ethnicity, education and income.

[c] Adjusted for age, sex, race/ethnicity, education, income, smoking, drinking, family history of cardiovascular disease and family history of diabetes.

*P < 0.05, **P < 0.01, ***P < 0.001.

© 2024 Zhang X et al. *JAMA Network Open.*

eFigure 1. Trends of Mean BRI Values in US Adults From the NHANES, 1999 to 2018.[a]

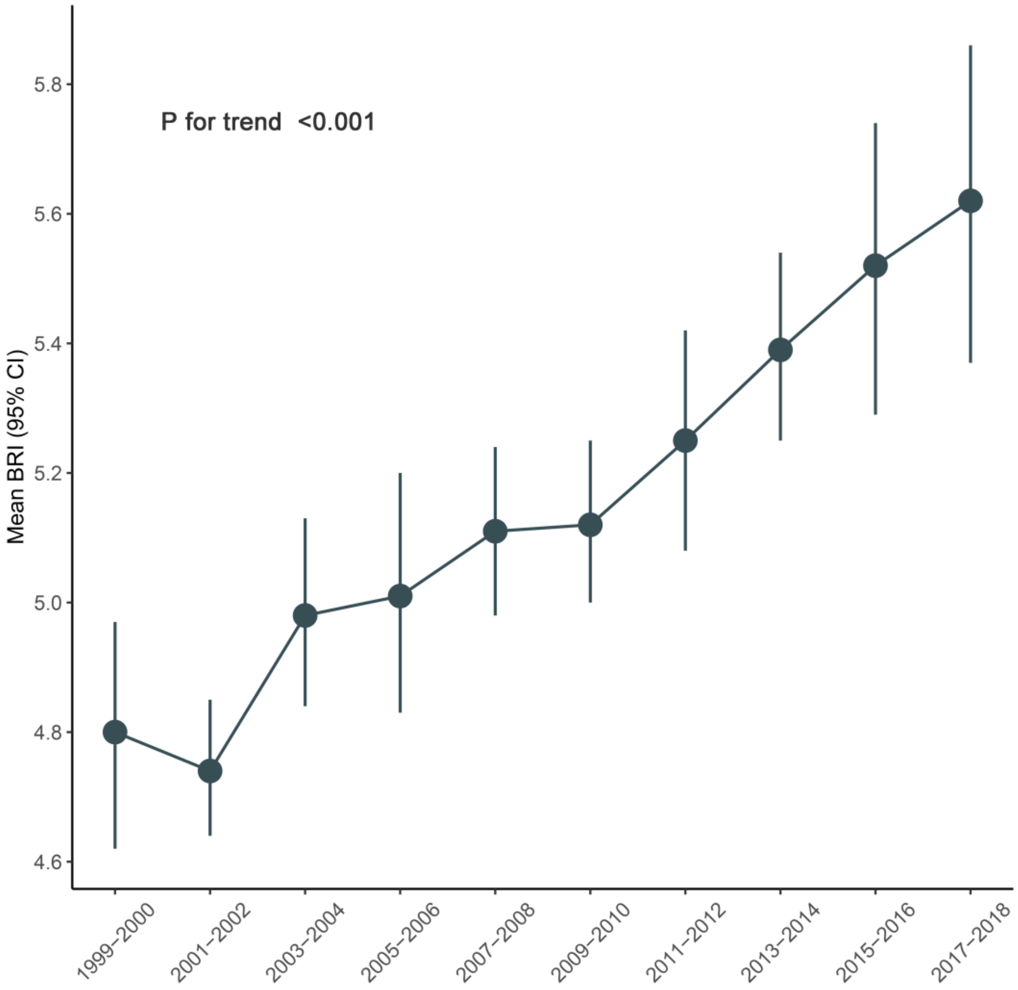

Abbreviations: BRI, body roundness index; NHANES, National Health and Nutrition Examination Survey; 95% CI, 95% confidence interval.

[a] Nationally representative estimates of the nonpregnant US population aged 20 years or more. Estimates were nationally representative through the use of survey weights.

Solid circle stands for mean BRI value of each NHANES cycle, and the upper and lower vertical extremes of each circles represents the corresponding 95% CI.

The y-axis does not necessarily start at 0 for clearer visualization of trends.

© 2024 Zhang X et al. *JAMA Network Open.*

eFigure 2. Trends of BRI by Socioeconomic Factors in US Adults From 1999 to 2018.

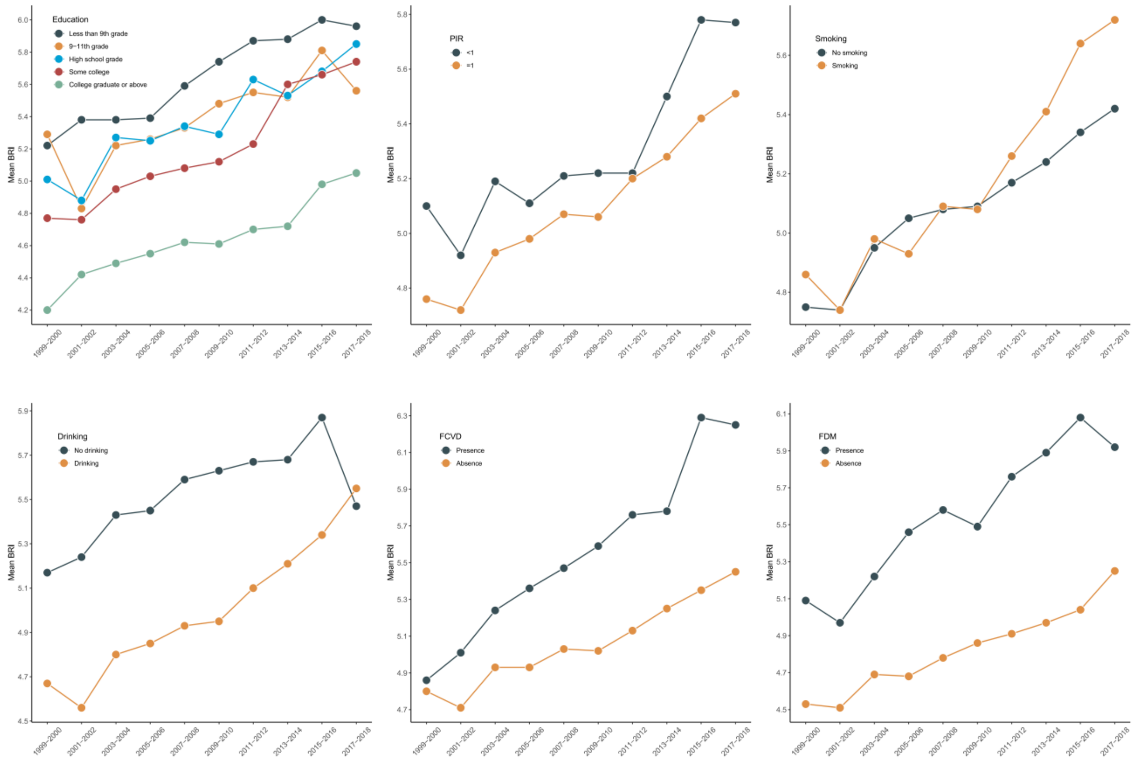

Abbreviations: BRI, body roundness index; PIR: poverty impact ratio; FCVD, family history of cardiovascular disease; FDM, family history of diabetes.

[a] Nationally representative estimates of the nonpregnant US population aged 20 years or more. Estimates were nationally representative through the use of survey weights.

The y-axis does not necessarily start at 0 for clearer visualization of trends. Solid circle stands for mean BRI value of each NHANES cycle.

© 2024 Zhang X et al. *JAMA Network Open.*

eFigure 3. The distribution plot of ln(-ln(S(t))) relative to ln(t) for assessing Cox assumption of body roundness index (BRI) in quintiles.

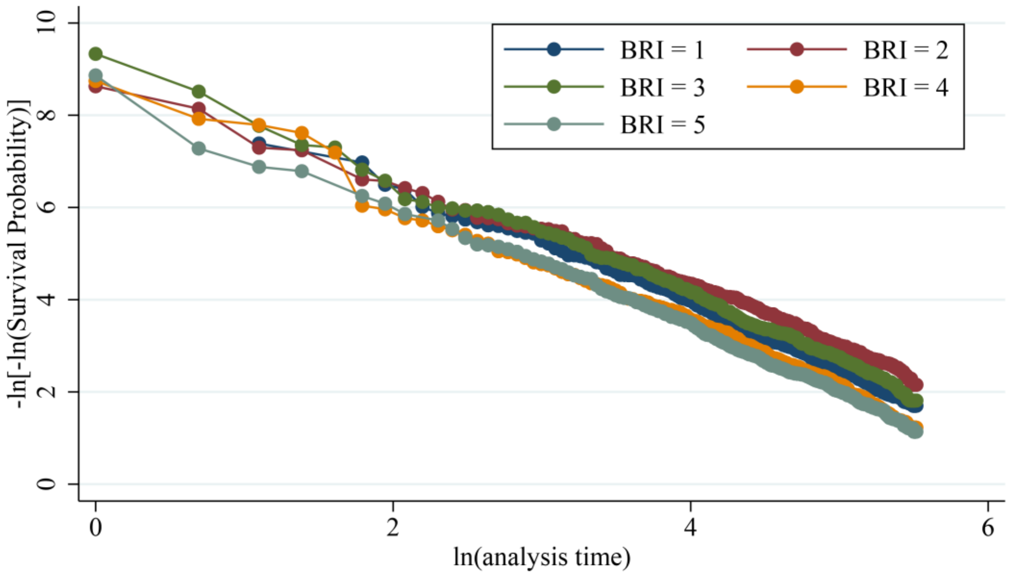

© 2024 Zhang X et al. *JAMA Network Open.*

eFigure 4. Adjusted HR of All-Cause Mortality by Sociodemographic Factors Comparing Quintile 1 (Q1) (Left Panel) and Quintile 5 (Q5) (Right Panel) with Quintile 3 (Q3) of BRI in US Adults from the NHANES Linked Mortality Files, 1999 to 2018, in Fully Adjusted Model.[a]

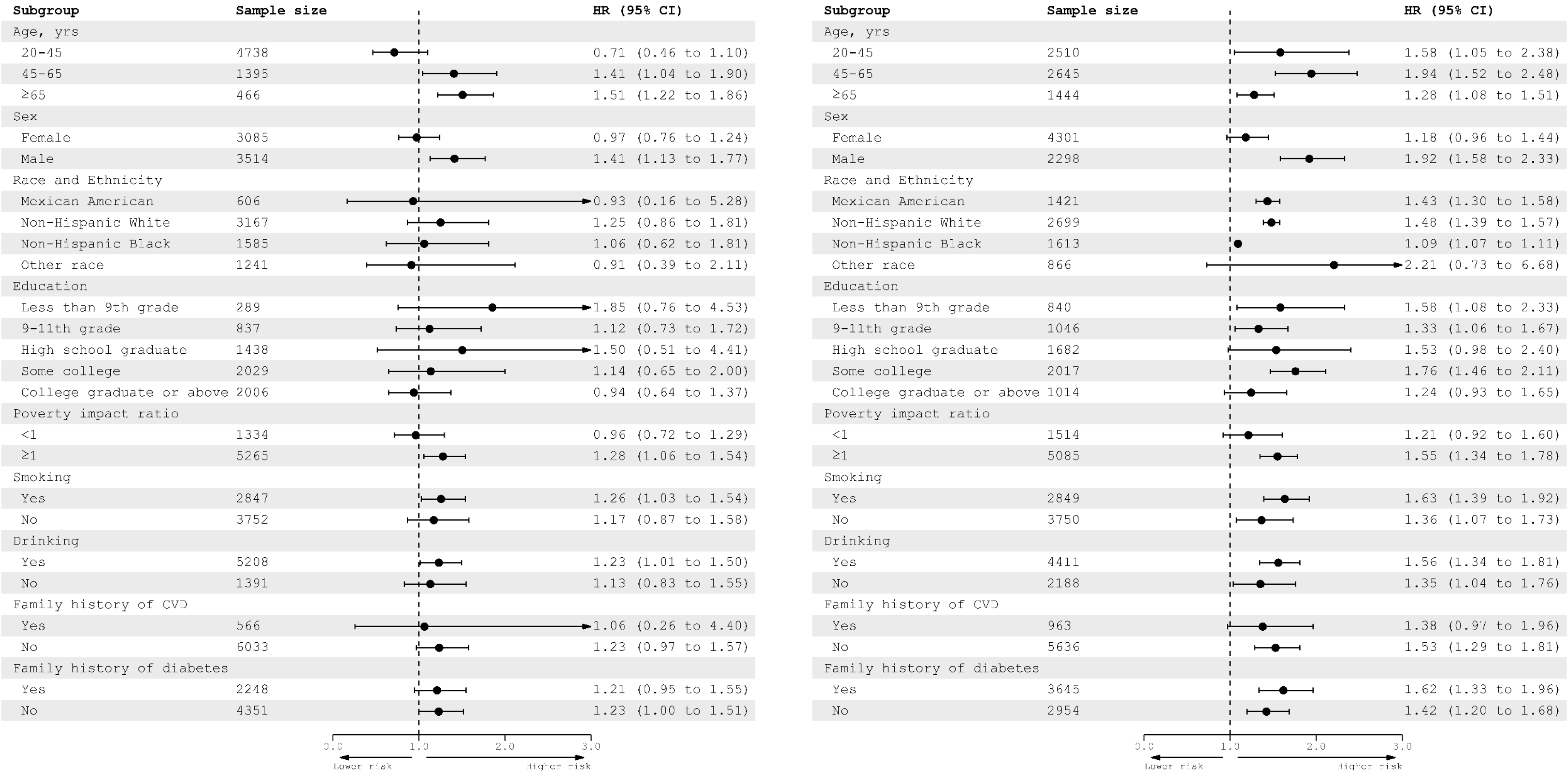

Abbreviations: BRI, body roundness index; NHANES, National Health and Nutrition Examination Survey; HR, hazard ratio; 95% CI, 95% confidence interval; CVD, cardiovascular disease.

[a] Estimates were nationally representative through the use of survey weights. HR and 95% CI were calculated after adjusting for age, sex, race/ethnicity, education, income, smoking, drinking, family history of cardiovascular disease, and family history of diabetes under the Weibull regression model.

[b] Other race included non-Hispanic Asian, American Indian or Alaska Native, and Native Hawaiian or Pacifc Islander.

© 2024 Zhang X et al. *JAMA Network Open.*

eFigure 5. Adjusted HR of All-Cause Mortality by Sociodemographic Factors Comparing Quintile 1 (Q1) With Quintile 3 (Q3) of BRI in US Adults From the NHANES Linked Mortality Files, 1999 to 2018, After Adjusting For Only Age and Sex (Left Panel),[a] and in Partially Adjusted Model (Right Panel).[b]

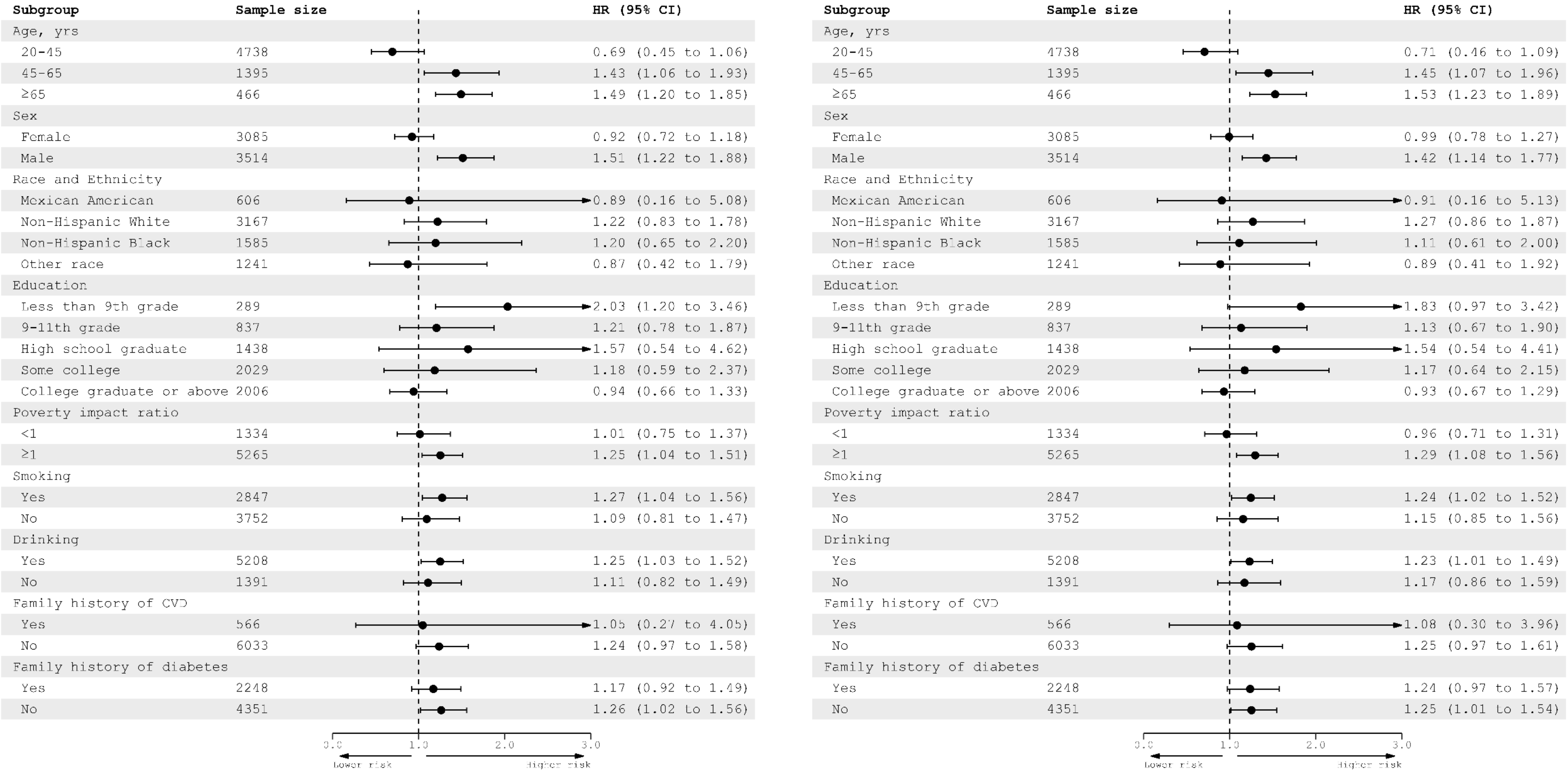

Abbreviations: BRI, body roundness index; NHANES, National Health and Nutrition Examination Survey; HR, hazard ratio; 95% CI, 95% confidence interval; CVD, cardiovascular disease.

[a] Estimates were nationally representative through the use of survey weights. HR and 95% CI were calculated after adjusting for age and sex under the Weibull regression model.

[b] Estimates were nationally representative through the use of survey weights. HR and 95% CI were calculated after adjusting for age, sex, race/ethnicity, education, and income under the Weibull regression model.

[c] Other race included non-Hispanic Asian, American Indian or Alaska Native, and Native Hawaiian or Pacifc Islander.

© 2024 Zhang X et al. *JAMA Network Open.*

eFigure 6. Adjusted HR of All-Cause Mortality by Sociodemographic Factors Comparing Quintile 1 (Q5) With Quintile 3 (Q3) of BRI in US Adults From the NHANES Linked Mortality Files, 1999 to 2018, After Adjusting For Only Age and Sex (Left Panel),[a] and in Partially Adjusted Model (Right Panel).[b]

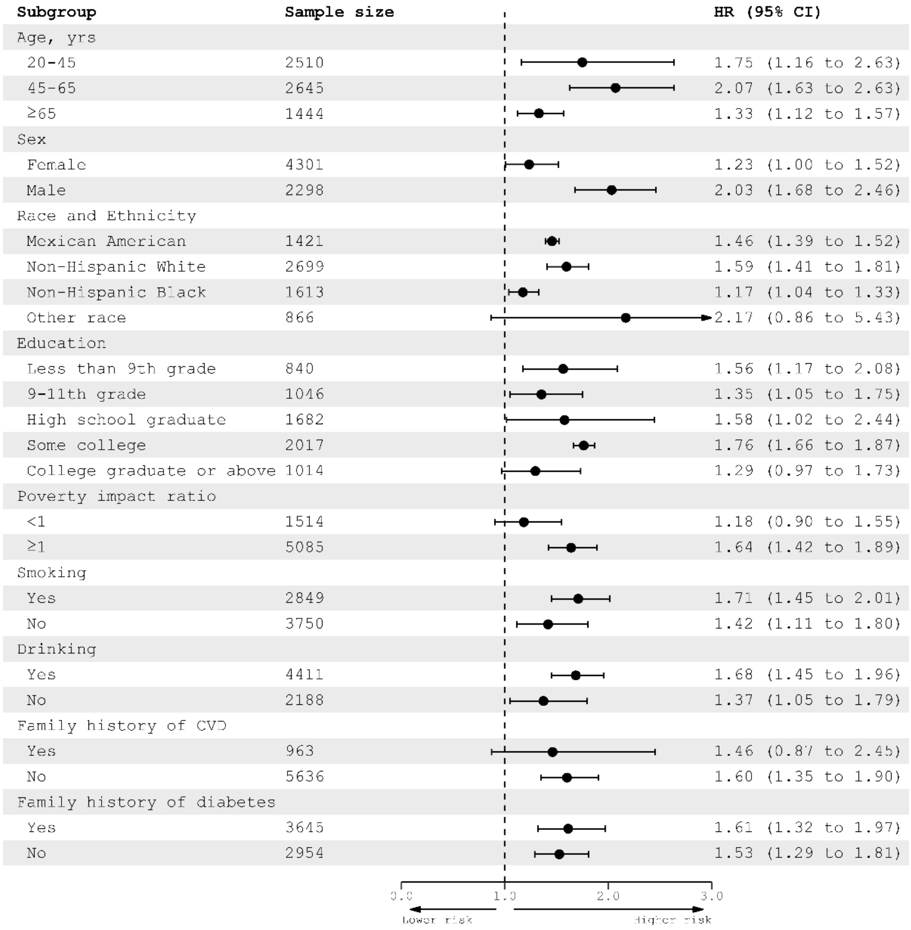

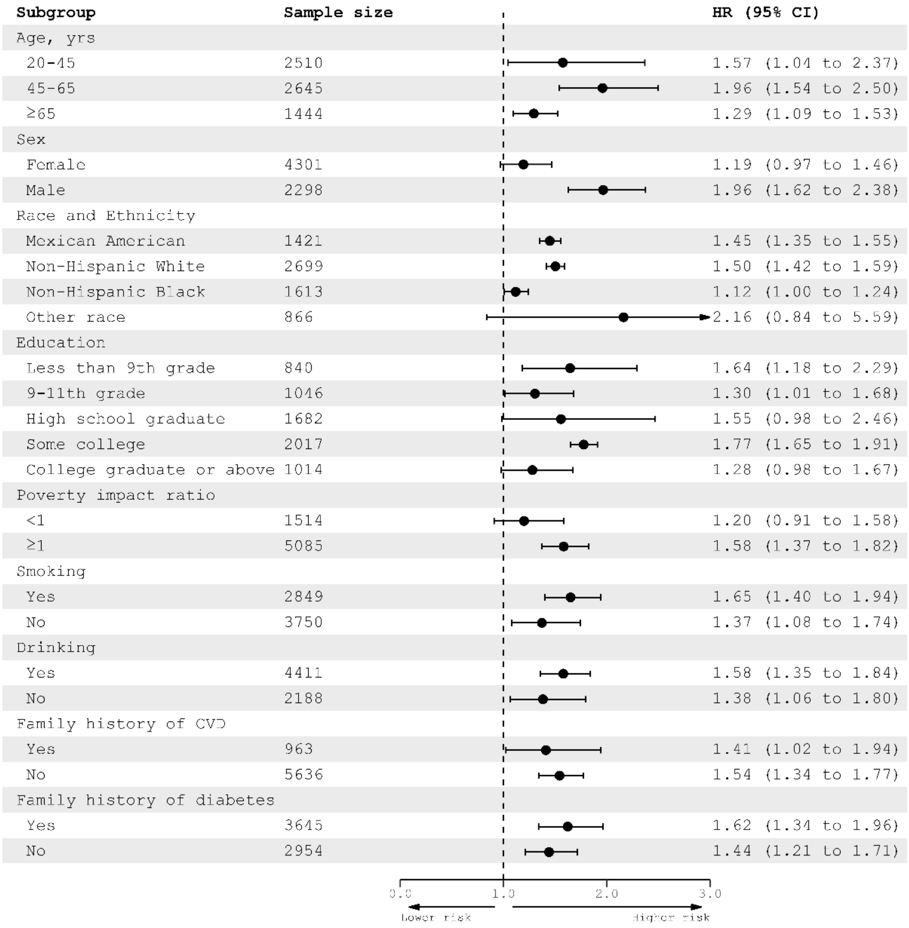

Abbreviations: BRI, body roundness index; NHANES, National Health and Nutrition Examination Survey; HR, hazard ratio; 95% CI, 95% confidence interval; CVD, cardiovascular disease.

[a] Estimates were nationally representative through the use of survey weights. HR and 95% CI were calculated after adjusting for age and sex under the Weibull regression model.

[b] Estimates were nationally representative through the use of survey weights. HR and 95% CI were calculated after adjusting for age, sex, race/ethnicity, education, and income under the Weibull regression model.

[c] Other race included non-Hispanic Asian, American Indian or Alaska Native, and Native Hawaiian or Pacifc Islander.

© 2024 Zhang X et al. *JAMA Network Open.*

eFigure 7. The relationship between body mass index and all-cause mortality risk after full adjustment.

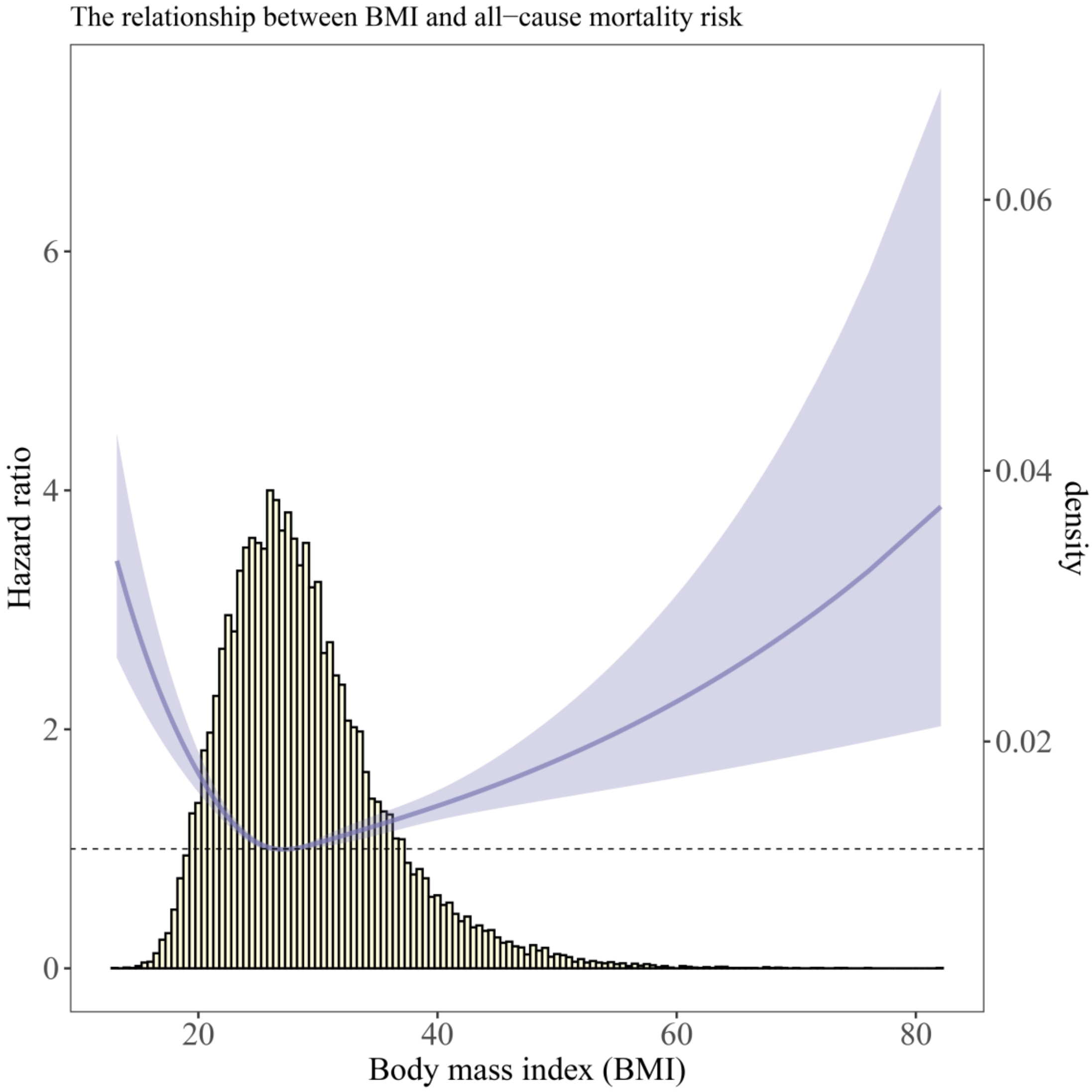

The solid curve line represents the effect-size estimates for the association of BMI with all-cause mortality, and the light shadow represents the 95% confidence interval.

© 2024 Zhang X et al. *JAMA Network Open.*
