# Supplementary material for: Body Roundness Index and All-Cause Mortality Among US Adults
Source: JAMA Netw Open. 2024 Jun 5;7(6):e2415051. doi: 10.1001/jamanetworkopen.2024.15051 (PMC11154161; doi:10.1001/jamanetworkopen.2024.15051)
Supplement: Supplement 2. — Data Sharing Statement [file jamanetwopen-e2415051-s002.pdf]

Data Sharing Statement

Zhang. Body Roundness Index and All-Cause Mortality Among US Adults. *JAMA Netw Open*. Published June 05, 2024. doi:10.1001/jamanetworkopen.2024.15051

Data
Data available: No
